# Implementation of palliative care for patients with terminal diseases from the viewpoint of healthcare personnel

**DOI:** 10.1186/s13104-019-4260-x

**Published:** 2019-04-08

**Authors:** Nita Arisanti, Elsa Pudji Setiawati Sasongko, Veranita Pandia, Dany Hilmanto

**Affiliations:** 1 Department of Public Health, Faculty of Medicine, Universitas Padjadjaran, Jalan Eykman No. 38, Bandung, 40161 Indonesia; 2 Department of Psychiatric, Faculty of Medicine, Universitas Padjadjaran, Jl. Pasteur No. 38, Bandung, 40161 Indonesia; 3 Department of Child Health, Faculty of Medicine, Universitas Padjadjaran, Jl. Pasteur No. 38, Bandung, 40161 Indonesia

**Keywords:** Healthcare personnel, Implementation, Palliative care, Qualitative research

## Abstract

**Objective:**

In Indonesia, palliative care has not been uniformly implemented at all levels of healthcare facilities. Healthcare personnel play an important role in providing that care. This study aimed to explore the current conditions and expectations regarding palliative care from the perspective of healthcare personnel.

**Results:**

A qualitative study was conducted with 12 physicians and five nurses from December 2017 to June 2018. In-depth interviews of these professionals were conducted. The responses were subjected to inductive thematic analysis, generating five themes and 24 subthemes. The themes were (1) family and environment, including barriers and contributions to palliative care; (2) numbers and competence of healthcare providers; (3) accessibility of palliative care; (4) case management of patient’s and family’s problems by healthcare personnel; and (5) barriers or enabling factors from the healthcare system. Patients, family members, and healthcare personnel contribute to case management. Attention must be paid to improving access and the healthcare system for thorough implementation of palliative care.

## Introduction

Palliative care was introduced into the Indonesian healthcare system in 1989. In 2007, the Indonesian government indicated their support by issuing the Palliative Care Policy, decreeing that palliative care can be implemented in several types of healthcare facilities. In fact, the implementation of palliative care is still very limited in certain hospitals [1]. Cultural and socioeconomic factors, perceptions of patients and their families, attitudes of healthcare providers, lack of education and training for healthcare personnel, uneven distribution of palliative care facilities, lack of coordination, and limited funds serve as barriers to implementation [2, 3].

Support for implementing palliative care requires competent healthcare providers, adequate facilities, and good public awareness. It will require collaboration between healthcare personnel in hospitals and primary care. Healthcare professionals play an important role and a positive effect in caring for patients with terminal disease [4]. Given the complexity of each patient’s health problems, those caring for them must be competent and qualified. A study noted that patients and families need staff who can provide psychological and spiritual support, assist in decision making, manage symptoms, and communicate well [5]. However, many healthcare personnel do not understand the concept and practice of palliative care [1, 3]. A common misconception is that palliative care is provided only for dying patients [1, 4]. As a result, many patients with terminal diseases are not managed in a timely fashion.

This study aimed to explore the current situation of and expectations for the implementation of palliative care from the perspective of healthcare personnel.

## Main text

### Methods

This was a qualitative research study that used a case study approach. This approach is useful when there is a need for in-depth exploration and understanding of an issue in the real-life context. Such case studies can explore causal links involved in the development of a new policy or service [6, 7]. The study was conducted in nine primary healthcare facilities with a high prevalence of chronic diseases and one secondary and one tertiary hospital in Bandung District. Bandung is the capital of the most populous province in Indonesia. The data were collected from December 2017 to June 2018.

#### Informants

The informants selected from the facilities included physicians and nurses experienced in caring for patients with terminal diseases. Eligible informants who had enough time and were willing to be interviewed were approached directly by the researcher (NA). Two individuals refused to be interviewed.

#### Data collection

The interviews were conducted with informants from different disciplines and backgrounds to capture a multitude of perspectives and contexts. In-depth interviews were conducted using interview guidelines and were digitally recorded. The aim was to explore (1) personal experiences with palliative care and (2) issues, challenges, and expectations for implementation in the future. A list of topics for the interview was developed based on the literature. Written informed consent was obtained from all informants.

#### Data analysis

Inductive thematic analysis was applied to the results of the interviews to generate themes. The interviews were transcribed verbatim, and NVivo 11.0 software was used to code the transcripts. Data analysis was performed by line-by-line coding of the transcripts with focus on the implementation of palliative care. Researchers coded independently and merged codes if needed. Similar codes were merged into subthemes and grouped into potential major themes. When theme saturation was reached, no further interviews were necessary. The triangulation method, checking data and members was applied to improve validity and reliability. The researchers reflexively evaluated their role in data collection and analysis. Data, codes, and themes were discussed within the research team, which included a family physician, public health practitioner, pediatrician, and psychiatrist. The researchers had debriefing sessions after the interviews.

### Results

Interviews were conducted with 17 healthcare professionals (Table 1). Most informants had pursued postgraduate training. On average, the completion of the interviews took 30–45 min.

**Table 1:** Characteristics of informants

No	Variable	N
1.	Gender	
	Men	4
	Female	13
2.	Education	
	Undergraduate training	5
	Postgraduate training	12
3.	Type of healthcare personnel	
	Physician in primary health care	6
	Physician in hospital	6
	Nurse in primary health care	3
	Nurse in hospital	2
4.	Role of healthcare personnel	
	Pediatrician	2
	Obstetrician and gynecologist	1
	Medical rehabilitation specialist	1
	Internal medicine specialist	1
	Family physician	4
	Medical doctor in secondary hospital	1
	Medical doctor in primary health care	2
	Nurse	5
5.	Type of service provider	
	Primary health care	9
	Secondary health care	1
	Tertiary health care	7

Five themes and 24 categories were identified from the interviews (Tables 2 and 3) to describe the existing situation and expectations of palliative care.

**Table 2:** Description of key themes

Themes	Discussion points
Family and environment	Refers to barriers and contributing factors to palliative care from family and environment
Numbers and competence of healthcare personnel	Refers to the numbers and competencies of healthcare personnel to deliver palliative care
Accessibility of palliative care	Refers to feasibility of getting palliative care
Case management	Refers to the management of patient’s and family’s problems by healthcare personnel
Healthcare system	Refers to barriers and enabling factors from healthcare system

**Table 3:** Themes and categories generated from the perspective of healthcare personnel

	Themes	Categories
Palliative care from the perspective of healthcare personnel	Family and environment	1. Empowerment 2. Perception and preference 3. Norm 4. Coping mechanism; and 5. Health-seeking behavior
	Numbers and competence of healthcare personnel	1. Empathy 2. Communication 3. Decision making; and 4. Self-confidence
	Accessibility of palliative care	1. Geographical access 2. Informational access; and 3. Socialization
	Case management	1. Case notification 2. Comprehensive care 3. Interdisciplinary collaboration 4. Integration of care; and 5. Follow-up
	Healthcare system	1. Policy 2. Standard and guideline 3. Availability of drugs 4. Networking 5. Financing 6. Quality assurance; and 7. Legal aspect

#### Contributions from family members and the environment

Subthemes relating to the family and environmental context identified issues that could hinder or enable implementation. These included (1) empowerment, (2) perceptions and preferences, (3) norms, (4) coping mechanisms, and (5) health-seeking behavior.

Informants stated that empowerment can encourage the family in caring for patients or providing support to the patient in dealing with problems.

*“certainly cannot take care of themselves, so it is important to teach and empower the patient and family to be able to take care of themselves.”*

Empowerment occurs when the patient and family members understand the problems. However, lack of understanding and family preferences may be barriers in obtaining palliative care. Most family members prefer to accept the patient’s condition rather than to seek palliative care.

*“In reality, … patient and the family are still perceiving that the patient’s disease can be cured. This condition becomes our problem in providing care.”*

#### Numbers and skills of healthcare personnel

Informants noted that there are not enough healthcare personnel adequately skilled to deliver palliative care. Needed skills emerging from the interviews were (1) empathy, (2) communication, (3) decision-making, and (4) self-confidence.

The informants indicated that communication and decision-making skills are important. Many cases were cited showing that doctors and other healthcare providers do not adequately explain the patient’s problems and palliative care plan.

*“Sometimes it also becomes a problem when we should decide a treatment plan…most families prefer to take care of the patients at home.”*

A lack of skills and self-confidence among healthcare personnel is identified as a barrier to optimal care.

*“…many doctors refuse to take care of patients because they are not confident…”*

#### Accessibility of care

The reasons many patients could not access palliative care, as noted by informants, were (1) geographical conditions, (2) lack of information, and (3) lack of socialization. Palliative care services are more often located in urban areas and are limited in number.

Informants highlighted the fact that many patients and healthcare providers still do not know about palliative care and how to obtain it, resulting in many patients not getting adequate care and perceiving the term “palliative care” negatively.

*“Maybe because they don’t know the reasons to be referred to palliative…A lot of people don’t know about palliative concept”*

#### Case management

Factors important in effectively managing patients are (1) case notification, (2) comprehensive care, (3) interdisciplinary collaboration, (4) integration of care, and (5) follow-up.

Case notification is important in determining whether the patient needs palliative care, so that care can be provided early in the course of the disease.

*“…most patients are referred to the palliative team in advance and critical condition… the patients should be screened to be able to admit the palliative care…”*

Continuity of care is needed to thoroughly address all problems of the patient. Informants stated that follow-ups for many patients were not done adequately because there was no referral process from the hospital to the primary care site.

*“Yes, the problem is the referral doesn’t work properly… ideally the patient is followed up when he is sent home”*

Some informants agreed that palliative care should be given early in the disease course, but in many cases, palliative teams are involved only at the end of the patient’s life.

*“Unfortunately, we have known the patient’s condition lately. If we had known from the beginning… it would have been better, but if it is too late what can we do?”*

Another important factor to palliative care is interdisciplinary collaboration.

*“… home visit for end of life care can be done by nurses or doctors in the primary care, but we may need to consult or coordinate when we have patient with complex diseases”*.

#### Healthcare system

Issues related to the healthcare system explored in the interviews consisted of (1) policy, (2) standard and guideline, (3) availability of drugs, (4) networking, (5) financing, (6) quality assurance, and (7) legal aspects.

A policy is expected to be implemented at all levels of healthcare by developing standard and guideline. Pain medications and the authority to prescribe them must be available, and legal protection for the providers should be regulated. Financing may be a problem because, from the informants’ perspective, some health insurance policies do not cover palliative care.

### Discussion

According to the perspectives of healthcare professionals interviewed for this study, support from the family is an important aspect for improving the quality of life of patients with terminal disease. These problems are rarely identified by the palliative care team [5, 8]. Many families do not understand the condition of their loved one and are afraid of the concept of palliative care. Palliative care is often associated with death [9–11], although most family members still believe that the disease can be cured. The understanding and perceptions of patients and their families are related to the norms and values of the family and social concerns [9, 10]. These values will affect their behavior, especially the health-seeking behavior.

Palliative care cannot be implemented in several healthcare facilities owing to the limited number of trained personnel. Ideally, healthcare professionals should have good communication skills and empathy to build good relationships with patients and families [12]. Patients and their families require detailed information about the disease and its management. Being able to communicate in a respectful and controlled manner in a comforting environment is important in providing care that will improve the quality of life [5, 13]. Another hindrance is that many physicians are reluctant to provide palliative care because they are not confident in their ability to do so. Being gentle and professional are skills that patients expect from a palliative care physician [10].

Comprehensive management via a personalized approach and regular follow-up are needed for patients with terminal disease. The aim is to eliminate problems and improve the quality of life. This requires involvement of multidisciplinary teams. Comprehensive care via an interdisciplinary approach from an early stage of the disease ensures appropriate disease management and links the care provided in the hospital, home, and hospice setting [14, 15]. The lack of such collaboration will result in ineffective services [16].

The informants agreed that palliative care must be integrated throughout the course of the patient’s disease. In some countries, palliative care may be available earlier in the disease trajectory [14, 17]. However, the integration needs to be tailored to the healthcare system in each country and the availability of resources [18]. At the policy level, the integration of palliative care into the healthcare system is essential for its implementation.

The absence of subsidies or health insurance and the high cost of care are major barriers to accessing palliative care [17]. Advocating for government or health financing agencies to include palliative care in health insurance schemes is necessary.

Primary care physicians noted difficulties in providing pain medication, especially for severe pain. This is owing to the limited availability and restrictions on the prescription of opioid drugs. This situation was also described by hospital-based informants. It results in some patients receiving inappropriate pain management. Improved access to opioid drugs and greater availability is essential for good pain management [19].

### Conclusion

Several factors are essential for the implementation of palliative care. Patients, family members, and healthcare personnel have contributions to make in disease management. Thorough implementation of palliative care also requires improvement in access to care and support of the healthcare system.

## Limitations

To our knowledge, this is the first study to explore the personal experiences of professionals, the challenges they face, and their expectations for improved implementation of palliative care in Indonesia. A limitation of the study is that it was conducted in one city. Future research could include several settings, such as Jakarta, Surabaya, and Yogyakarta, and explore the wider perspectives of healthcare personnel on the implementation of palliative care.
